# Cryopreservation of Ovarian Tissue in Pediatric Patients

**DOI:** 10.1155/2012/910698

**Published:** 2012-02-13

**Authors:** R. Fabbri, R. Vicenti, M. Macciocca, G. Pasquinelli, M. Lima, I. Parazza, V. Magnani, S. Venturoli

**Affiliations:** ^1^Gynecology and Reproductive Medicine Unit, Department of Obstetrics and Gynecology, S. Orsola-Malpighi Hospital, Bologna 40138, Italy; ^2^Surgical Pathology Unit, Department of Haematology, Oncology and Clinical Pathology, S. Orsola-Malpighi Hospital, University of Bologna, Bologna 40138, Italy; ^3^Department of Pediatric Surgery, S. Orsola-Malpighi Hospital, University of Bologna, Bologna 40138, Italy

## Abstract

Cancer treatments improve the survival rate of children and adolescents; however chemo- and radiotherapy result in gonadal damage leading to acute ovarian failure and sterility. Ovarian tissue cryopreservation allows long-term storage of primordial follicles and represents the only possibility of preserving the potential fertility in prepubertal girls. The aim of the present study is to describe our experience in ovarian tissue cryopreservation in 45 pediatric patients. The number of follicles per square millimeter of the overall section area and follicle quality were evaluated histologically. A strong negative correlation was found between age and follicular density in patients both prior to and after chemotherapy (*P* < 0.0001). Damage in follicular quality, that is, increased oocyte vacuolization and detachment of the oocyte from granulosa cells, was found after chemotherapy. Ovarian tissue cryopreservation, preferably performed before initiation of chemotherapy, should be offered to pediatric patients, including prepubertal girls, at risk of sterility.

## 1. Introduction

The survival rate of children and adolescents with cancer has increased markedly over the past 30 years as a result of advances in supportive care and changes in cancer therapies. The use of cancer multimodal therapy (e.g., surgery, radiotherapy, and intensive multiagent chemotherapy) is now routine in the treatment of children with neoplastic disease. However many children treated for cancer suffer from side-effects of curative chemo- and radiotherapy, such as the risk of Acute Ovarian Failure (AOF) or early menopause, then sterility [1]. Gonadal damage results in a development arrest of secondary sexual characteristics that prevent the attainment of sexual maturity. As the majority of women who survive cancer in childhood expect a normal reproductive life span, an early loss of fertility can seriously harm their quality of life.

The level of ovarian damage varies according to the cancer treatment protocol: ovarian integrity is affected by the type of chemotherapeutic agent, the cumulative dose of chemotherapeutic agents, treatment duration, and total dosage effect. In general alkylating agents such as cyclophosphamide are associated with the highest risk of infertility [2–5]. Ovarian failure has also been reported in patients treated with other agents such as cisplatin and vinca alkaloids. Of the multiagent treatment protocols, the adriamycin (doxorubicin), bleomycin, vinblastine, and dacarbazine (ABVD) combination is considered less cytotoxic than other protocols. Furthermore, the prebone marrow transplantation (BMT) conditioning protocol of busulfan and cyclophosphamide has been found to lead to high rates of sterilization, whereas high-dose melfalan seems to be safer [2].

Previous studies indicate that radiation affects the ovaries and the degree of impairment depends on the radiation dose, fractionation schedule, and radiation field. Doses as low as 400–600 cGy in adults and 1000–2000 cGy in children have been noted to decrease ovarian function [6]. The risk is higher with increasing doses of radiation. Radiotoxicity appears to be higher when radiation is given as a single dose as opposed to fractionated. Irradiation to abdominal, pelvic, or spinal regions is associated with increased risk for AOF [7]. However, even scattered radiation, not directed specifically to the pelvis or abdomen, can cause ovarian damage. Patients treated with total body irradiation before BMT together with high doses of chemotherapeutic agents are very likely to have severe ovarian dysfunction [2].

The most well-established options for female adult fertility preservation are embryo and oocyte cryopreservation. Unfortunately, in the pediatric setting embryo cryopreservation is inappropriate and ovarian tissue cryopreservation maybe an alternative option for fertility preservation in these patients: with ovarian tissue freezing, no ovarian stimulation is needed and ovarian tissue can be harvested laparoscopically without any preparation. Furthermore ovarian tissue cryopreservation has the greatest fertility potential in children since they have a high number of primordial follicles [4, 7–15]. 

After thawing, cryopreserved ovarian tissue can be grafted into its normal anatomical position or into a heterotopic site. Alternatively the thawed tissue can be placed in culture with the aim to achieve antral follicle development and to avoid the risk of reimplantation of malignant cells [16]. However this option has not so far progressed sufficiently to be a therapeutic possibility [12].

Until today all pregnancies obtained after transplantation of frozen-thawed ovarian cortex were observed in adult patients at the time of harvesting; however there is no reason to doubt the capacity of prepubertal ovarian cortex to develop and function correctly after reimplantation [14]. Animal studies have demonstrated that puberty and cyclic hormonal activity can be restored by reimplantation of fresh and frozen–thawed prepubertal ovarian tissue in both prepubertal and adult mice [17, 18]. In humans only one experience has been reported by Andersen (data shown to the ESHRE Campus Symposium, Fertility Preservation in Cancer, Bologna, 2010). The patient was affected by Ewing Sarcoma at the age of 9 years, and her ovarian tissue was cryopreserved before receiving chemo- and radiotherapy. After cancer treatment, the patient presented clinical and biological Premature Ovarian Failure (POF) and, at the age of 13, she underwent reimplantation of cryopreserved ovarian tissue. One year later, the patient had her first menstrual cycle and evident signs of pubertal development. However, the uterus of the patient did not grow to adult size, perhaps because the pelvis had received high doses of irradiation. It is well known that pelvic radiotherapy decreases uterine blood flow and reduces uterine volume and endometrial thickness [3, 4, 19].

The purpose of this paper is to describe our experience in ovarian tissue cryopreservation in pediatric patients focusing on the clinical characteristics of the patients, the technical procedures, and strategies used to harvest and preserve the tissue. In adolescent patients, the opportunity to preserve their fertility enabling a future normal life is a psychological support allowing the patients to overcome the difficulties that the disease entails.

## 2. Materials and Methods

### 2.1. Subjects

From January 1999 to August 2011, 51 pediatric patients with malignant and benign diseases approached the Gynecology and Reproductive Medicine Unit, Department of Obstetrics and Gynecology, S. Orsola-Malpighi Hospital, Bologna, for consultation about the possibility of ovarian tissue cryopreservation. The ovarian tissue of 45 pediatric patients, including pre-and postmenarcheal girls, was collected and cryopreserved. Parents of the patients gave their consent for both laparoscopy and cryopreservation of ovarian tissue. Ovarian tissue cryopreservation was not carried out for six patients: in two cases patients did not fit the inclusion criteria; in two cases parents refused because the procedure was too new or they did not want their daughter to be subject to further surgery; in the last two cases the patients suffered from Turner Syndrome with very small ovaries and a menopausal value of FSH, so, given the possibility of cryopreservation of few follicles, their parents refused consent.

At our centre, the exclusion criteria for ovarian tissue cryopreservation included patients positive for Hepatitis B Virus (HBV), Hepatitis C Virus (HCV), Human Immunodeficiency Virus (HIV), or Treponema Pallidum (TP); patients with ovarian cancers; and patients who had contraindications for surgery. Regarding patients with leukaemia could be removed and cryopreserved in the hope that in vitro maturation of the oocytes will be possible in the future [3]. 

As listed in Table 1, the majority of patients sent to our centre were cancer patients (38 of 45); others were affected by primary or genetic bone marrow disorders (6 of 45) and autoimmune disorders (one of 45). Within the cancer patients, more than half (23 of 38) had completed one or more cycles of chemotherapy before the collection of ovarian tissue (Table 1). According to the Karnofsky Performance Status Scale Index, the patients enrolled in the study reached over 90% in the criteria scale.

Ovarian tissue cryopreservation was carried out within a maximum of 20–30 days from the last chemotherapy cycle, while within 10–15 days in patients not undergoing chemotherapy before ovarian tissue recovery. The ovarian preservation procedure was not associated with any delay in the commencement of anticancer treatment. 

### 2.2. Surgical Procedure

Ovarian tissue was collected at our department by laparoscopy. In all cases laparoscopy was carried out as a day surgery procedure under general anesthesia. A carbon dioxide pneumoperitoneum at a pressure of 10 mmHg was created via a Verres needle inserted into the peritoneal cavity through an umbilical incision. A three-port laparoscopy was performed. The primary 5 mm port was inserted through the umbilicus for the telescope; two secondary 3 mm or 5 mm ports were inserted to the left and the right, respectively, lower quadrants at the same level lateral to the rectus sheath. Ovarian tissue was removed with scissors proceeding along the connecting line between two atraumatic graspers positioned in the ovary just above the infundibulopelvic and utero-ovarian ligaments first on the left and then on the right. Each ovarian fragment was placed in an Endobag (Cook Medical) and retrieved through the umbilical 5 mm port to avoid tissue damage. The harvested tissue was then transferred to the laboratory for immediate cryopreservation. In the majority of cases, there was negligible bleeding of the ovaries; haemostasis was carried out by bipolar current. The port sites were closed using 4–0 resorbable (Biosyn) sutures. The median duration of this technique was 30 minutes (range: 15–45 min). All patients were discharged on the day of surgery or at most the day after, with the discomfort experienced being no greater than after diagnostic laparoscopy. No complications occurred due to anesthesia or surgery in any patient.

Forty-three patients underwent laparoscopic harvesting of ovarian tissue from both ovaries, while in two patients the ovarian tissue was harvested from only one ovary because the other ovary was too small (patient with Wilms Tumor) and in the other case unilateral ovariectomy was performed (patient with Hodgkin Lymphoma). The amount of ovarian tissue removed was subordinated to the anticancer treatment protocol and to the size of the ovaries: in premenarcheal patients, due to the small size of the ovary and a high density of primordial follicles, the biopsy was smaller compared to the postmenarcheal ones.

### 2.3. Ovarian Transposition

When pelvic radiotherapy is indicated, ovarian transposition can be performed in order to displace the ovaries away from the radiation field [3–5, 20]. In our experience, we reported a combined approach of ovarian tissue cryopreservation and ovarian transposition in four patients (*n* = 19, Table 2; *n* = 9, 10 and 17, Table 3). In these cases the laparoscopy was carried out as a standard procedure. After ovarian tissue removal for immediate cryopreservation, the residual ovaries were temporarily transposed to the lower anterolateral abdominal wall without transection of the ovarian ligament and mesovarium. In particular, they were fixed intracorporeally to the peritoneum close to the ipsilateral round ligament of the uterus, using one 2–0 resorbable braided (Vicryl) continuous suture [21]. Ovarian transposition induces no particular injuries on the ovary per se, or to the blood supply vessels, with no adhesions and disruption of anatomical relationships, indicating that it may be a valid strategy for the preservation of ovarian function [21].

### 2.4. Cryopreservation Procedure of Ovarian Tissue

The cryopreservation protocol used by our group was approved by the Ethics Committee of S. Orsola-Malpighi Hospital (Clinical trial 74/2001/0 approved on 13/02/2002).

After biopsy, the ovarian tissue was placed in Dulbecco's phosphate-buffered solution (PBS) (Gibco, Life Technologies LTD, Paisley, Scotland) with 10% human serum added (HS was provided by the Transfusion Centre of S. Orsola-Malpighi Hospital) and cut into pieces (mean size 1 × 0.3 × 0.1 cm) using a scalpel. The mean number of pieces obtained was 23.1 ± 6.8 (mean ± SE) in premenarcheal patients and 27.6 ± 2.9 (mean ± SE) in postmenarcheal patients. One or two pieces (1–3 mm) from each ovarian sample were immediately fixed for histological analysis. The remaining tissue was maintained in PBS + 10% HS solution until cryopreservation. Samples were cryopreserved using a slow-freezing/rapid-thawing protocol, according to Fabbri et al. [22]. At the end of the cooling program, the cryovials were transferred into liquid nitrogen and stored until thawing. 

For each patient, a cryopreserved sample from each ovary was thawed after one month and fixed for histological analysis in order to evaluate the preservation of cortical tissue.

### 2.5. Histological Analysis

Fresh and frozen/thawed tissue samples were fixed in a freshly prepared solution of 2.5% glutaraldehyde in 0.1 mol/L sodium cacodylate buffer, pH 7.4, overnight at 4°C. After osmium tetroxide postfixation and alcohol dehydration, the samples were embedded in Araldite epoxy resin (Fluka, Buchs, Switzerland) and then sectioned with an ultramicrotome (Ultracut; Reichert, Wien, Austria). For each sample one 0.5 *μ*m thick section out of every 50 was collected and stained with toluidine blue for light microscopy (LM) examination (Leitz Diaplan light microscope equipped with a CCD JVC video camera). Semithin sections were examined, double blind by two trained pathologists, to (i) identify and count follicles, (ii) establish the number of damaged follicles, and (iii) assess follicular density, expressed as the number of follicles per square millimeter of the overall section area. Measurements were performed on digitalized images using ImageProPlus software. 

Semithin stained sections were initially observed at 10x to determine whether tissue was appropriate for histological examination: artefacts, erroneous sampling, presence or absence of follicles, and pathological involvement of ovarian tissue were recorded. Sections were then observed at medium (25x) and high magnification (40x). Follicles were counted and classified as resting type (primordial, intermediary primordial follicles, and small primary), growing type (large primary, secondary, preantral, antral), and atretic type, according to Gougeon classification [23]. At 40x the quality of follicles was determined; follicles containing oocytes with empty appearance, large cytoplasmic vacuoles, dark and granular cytoplasm, hyperchromatic nuclear staining, and detachment of the oocyte from the granulosa cells were considered degenerated. 

### 2.6. Hormone Assays

Blood samples were analyzed for FSH, LH, Estradiol, Estrone, Prolactin, Testosterone, and 17-Hydroxyprogesterone one or two days before laparoscopy. 

### 2.7. Follow-Up

Follow-up was performed six months after the end of therapy, and then every year, using ultrasound and hormonal tests.

### 2.8. Statistical Analysis

We studied the follicular density of the pre- and post-chemotherapy groups and, within each group, the follicular density of pre and postmenarcheal patients. The relationship between age and follicular density was evaluated using a linear regression model. Follicular density value used for statistical analysis was obtained by averaging the follicular densities of the left and right ovary. Results were statistically analysed by unpaired *t-*test. A value of *P* < 0.05 was accepted as significantly different. 

## 3. Results and Discussion

Ovarian tissue was collected and cryopreserved from 45 pediatric patients aged 160.9 ± 6.9 months (mean ± SE). Patients were grouped into two categories: pre-chemotherapy patients (*n* = 22) aged 53–214 months (161.4 ± 8.9, mean  ± SE) (Table 2) and post-chemotherapy patients (*n* = 23) aged 19–215 months (156.4 ± 10.6, mean ± SE) (Table 3). The specific chemotherapy regimen for each patient in relationship to the individual disease is given in Table 3.

In both groups, menarcheal age was 150 ± 3.8 months (mean ± SE). In the pre-chemotherapy group 80% of patients had regular menstrual cycles and normal flow, whereas 20% had slightly irregular menstrual cycles of up to 35 days. In post-chemotherapy patients, it was not possible to evaluate menstrual cycles because they had been treated with pills or GnRH analogues. 

A total of 5749 follicles from pre-chemotherapy patients and 2684 follicles from post-chemotherapy patients were analyzed; in both series LM showed that 99% of follicles were of the resting type and 1% distributed among primary, secondary, and preantral follicles. In one patient (*n* = 2, Table 3), the ovarian biopsy was too small and the entire cortex was cryopreserved. In all patients, LM on serial sections did not identify any neoplastic cells. Damaged follicles were observed in 26.1 ±  4.2% (mean ± SE) of the pre-chemotherapy group and in 34.3 ± 5.3% (mean ± SE) of the post-chemotherapy group (NS). The main alterations of damaged follicles observed were oocyte vacuolization and detachment of the oocyte from the granulosa cells. The high percentage of follicle damage in the pre-chemotherapy group could be attributed to the large number of patients with Hodgkin Lymphoma enrolled in this study. As shown in our study (Fabbri et al., 2011) [24], Hodgkin Lymphoma patients have a high frequency of vacuolization in resting oocytes (73.7%) before chemotherapy that positively correlates with disease stage, whereas in the control group only the 5.7% of the oocytes showed microvacuolar changes and large-sized vacuoles were never seen. In the post-chemotherapy group, the highest percentage of follicular damage confirmed a previous study demonstrating the gonadotoxic effect of chemotherapy on follicular quality [2, 25]. However the lack of significant difference in the percentage of damaged follicles between pre- and post-chemotherapy groups could be due to the great variability of chemotherapic protocol and to the low number of cycles performed in this group of patients. It is therefore possible that chemotherapy has not yet fully demonstrated its real injury to the ovary. 

**Figure 1:**
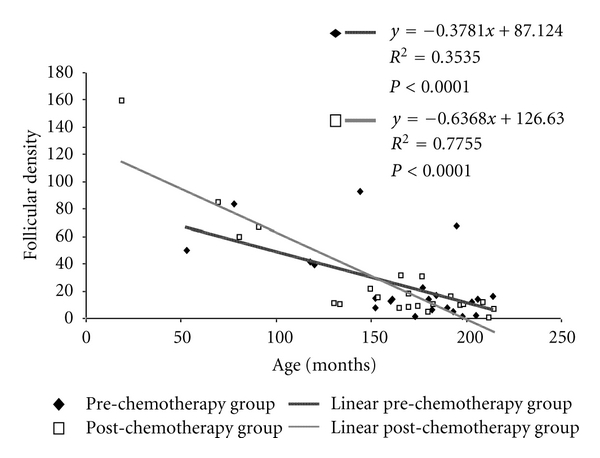
Correlation between age and follicular density in pre-chemotherapy and post-chemotherapy groups.

**Figure 2:**
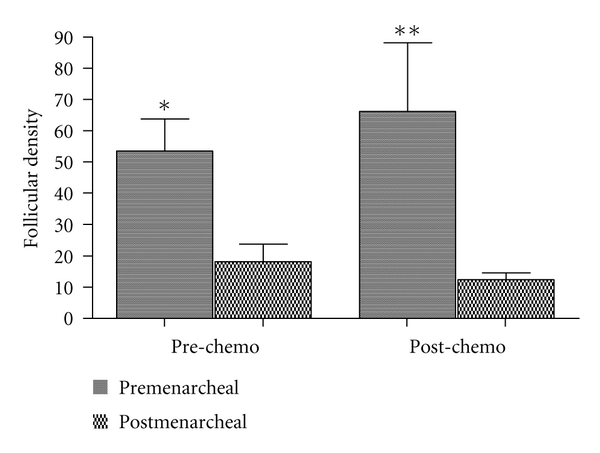
Comparison of follicular densities between premenarcheal and postmenarcheal patients in pre- and post-chemotherapy groups. *P < 0.05; **P < 0.001.

In agreement with literature [10, 26], we found an age-dependent decline in follicular density in pediatric patients. In the pre-chemotherapy group as well as in the post-chemotherapy group, the follicular density showed a statistically negative correlation dependent on patient age (*y* = −0.378*x* + 87.124, *R* ^2^ = 0.354, *P* < 0.0001; *y* = −0.637*x* + 126.63, *R* ^2^ = 0.776; *P* < 0.0001) (Figure 1). Follicular density results were higher in premenarcheal patients than in the postmenarcheal in the pre-chemotherapy group (53.4 ± 10.3 versus 18.2 ± 5.6, *P* < 0.05; mean ± SE) as well as in the post-chemotherapy group (66.1 ± 22 versus 12.4 ± 2.1, *P* < 0.001; mean ± SE) (Figure 2).

**Figure 3:**
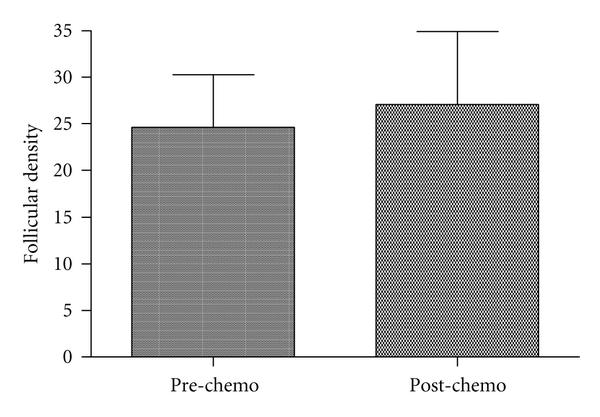
Comparison of follicular densities between pre-chemotherapy and post-chemotherapy groups. The difference is not significant.

Follicular density results were slightly higher in the post-chemotherapy group than in the pre-chemotherapy group (24.6 ± 5.7 versus 27.1 ± 7.8, NS; mean ± SE) (Figure 3). This finding may be due to the inclusion of the youngest patient (*n* = 1, Table 3 19 months) with the highest follicular density (159.16) in the post-chemotherapy group. 

Hormone levels of pre-chemotherapy patients were within the reference range of our laboratory for age and phase of menstrual cycle. Hormone levels of post-chemotherapy patients were not considerable because these patients were receiving pills or GnRH analogue treatment to protect the ovarian function.

Median follow-up time was 30 months (range 10–50 months). Thirty-six of the girls are still alive. Nine patients died of the primary pathology during the study period: three of these patients suffered from Acute Leukaemia, two from Hodgkin's Lymphoma, one from Wilms Tumor, one from PNET, one from Thalassemia Major, and one from Synovial Sarcoma. To date we have not received any request for ovarian tissue reimplantation because most of the patients are very young, many are still undergoing treatments, and others do not currently need or desire motherhood.

Ovarian tissue cryopreservation offers great promise for fertility preservation among young patients affected by cancer and represents the only option for prepubertal girls. The human ovary has a fixed number of primordial follicles which progressively decrease with increasing age in a biexponential fashion, culminating in the menopause at around 50 years of age. Both chemotherapy and radiotherapy quicken follicle depletion leading to premature menopause [27]. Ovarian tissue cryopreservation is particularly important in children because they have a high number of primordial and primary follicles that are particularly resistant to freezing and thawing protocols. A further benefit for pediatric patients is the possibility of using the cryopreserved ovarian tissue after a longer period than with adult patients. The probability of restoring fertility is related to the number and quality of primordial follicles in the ovarian cortical transplanted. Ovarian tissue cryopreservation also allows the restoration of steroidogenic function and promotes physiological development, puberty induction, and consequently the uterus growth. The puberty induction should be the main aim of ovarian tissue cryopreservation in prepubertal patients; alternatively these patients may undergo hormone replacement therapy and use the frozen ovarian tissue only if there is desire for motherhood. The final decision can only be made together by the oncologists, specialists in reproductive medicine and the parents who must make decisions based on the assumed future wishes of the girl. 

To date many authors have written about availability, feasibility, safety, and efficiency of ovarian tissue cryopreservation in pediatric patients. Jadoul et al. [14] performed ovarian cryopreservation in 58 girls under 16 years of age in order to validate the technique, concluding that ovarian cortex cryopreservation is feasible and as safe as comparable operative procedure in children. Poirot et al. [10] conducted a study to describe their experience of ovarian tissue cryopreservation in 49 prepubertal females before chemotherapy, focusing on the feasibility of the technique and specific features of the procedure for prepubertal children. They concluded that this procedure could be systematically offered to prepubertal girls at risk of sterility due to gonadotoxic treatment. Revel et al. [13] performed a study in which all patients undergoing ovarian cryopreservation were subject to integrated oocyte aspiration from antral follicle of the tissue, followed by In Vitro Maturation and oocyte cryopreservation as an additional fertility preservation strategy. They investigated the success of this procedure in 19 patients, aged 5–20 years, and retrieved oocytes from even the youngest patients (5- and 8-year-old girls). They concluded that patients undergoing ovarian cryopreservation could benefit from supplementary oocyte aspiration from the cortex. However being an experimental approach, it should be cautiously presented to young patients. Oktay and Oktem [7] reported the long-term follow-up and their experience with ovarian tissue cryopreservation in a series of 26 patients aged between 4 and 21 years of age, between 1999 and 2008. Their analysis suggested that tissue freezing in patients with different diseases and ages could be performed without significant risk. The study also revealed that the utilization rate of banked tissue is low, because the technology is only a decade old and because most patients undergoing ovarian cryopreservation are young and many are still undergoing treatment. Weintraub et al. [11] reported their experience on an ovarian cortex bank and presented a multidisciplinary discussion of the risks and benefits associated with ovarian tissue cryopreservation, suggesting that all girls about to receive treatment with a high risk of infertility be offered consultation on the possibility of performing ovarian cryopreservation. Anderson et al. [12] reviewed the advantages and disadvantages of ovarian tissue cryopreservation in the context of their experience of 36 women, 15 of whom were aged 16 or less. They highlighted the uncertainties surrounding the development of criteria for patient selection, the effects of chemotherapy on fertility, the most appropriate surgical techniques, and how many women will return to use their stored tissue. According to Borgstrom et al. [28], even patients with Turner Syndrome may benefit from cryopreservation as long as they exhibit essential characteristics for the recovering of follicles in the ovaries. In this study the authors involved 57 girls aged 8–19,8 with Turner Syndrome in order to evaluate which girls might benefit from ovarian tissue freezing for fertility preservation. They established five characteristics as being important for finding follicles in the ovaries of girls with Turner Syndrome: karyotype, low FSH levels, high AMH, spontaneous menarche, and spontaneous onset of puberty.

## 4. Conclusions

This study reports on a large number of pediatric girls undergoing ovarian tissue cryopreservation to preserve fertility. Our data show a high correlation between follicular density and age, and a decrease in follicular quality after chemotherapy. In light of these results and those reported in literature, the orientation of our group is in favour of ovarian tissue cryopreservation in pediatric patients because the procedure has a high potential of maintaining endocrine function and preserving fertility. It is well known that the optimal timing of ovarian cryopreservation is before sterilizing treatment. However, the live births obtained in adults after transplantation of ovarian tissue cryopreserved after chemotherapy and the high number of healthy follicles found in histological sections of tissue after chemotherapy in pediatric patients indicate that ovarian tissue cryopreservation could be offered after chemotherapy to young girls. In addition, the indication for fertility preservation in children can be extended to patients genetically predisposed to premature ovarian failure (e.g., Turner Syndrome), with acceptable results. Although no births have yet resulted from freeze-thawing of prepubertal ovarian cortex, expectations of this approach are encouraging, considering the high follicular pools of these patients.

## Figures and Tables

**Table 1:** Subjects.

Pathology	No. patients^a^	No. patients underwent chemotherapy^b^	Pathology	No. patients^a^	No. patients underwent chemotherapy^b^
Acute lymphoblastic leukaemia	5	5	Synovial sarcoma	1	1
Acute myeloide leukaemia	2	2	Ewing sarcoma	7	1
Hodgkin lymphoma	16	10	Myelodisplasia	1	
Non-Hodgkin lymphoma	1		Bone marrow aplasia	2	
Wilms tumor	3	2	Systemic lupus erythematosus	1	
Peripheral neuroectodermal tumor (PNET)	1	1	Thalassemia major	2	
Mixoide tumor	1		Medulloblastoma	1	1
Shwachman syndrome	1				

^a^ Total subject number enrolled in the study.

^b^Subject number underwent chemotherapy beforesurgery.

**Table 2:** Characteristics of the pre-chemotherapy patients who had ovarian tissue cryopreservation.

No.	Pathology	Age (months)	D/mm^2^ (RO)	D/mm^2^ (LO)	Cryopreserved fragments No.
1	Wilms tumor	53	49.89	—	63
2	Shwachmann syndrome	118	58.83	24.05	10
3	Ewing sarcoma	78	36.92	39.04	10
4	Ewing sarcoma	120	64.02	102.77	15
5	Hodgkin lymphoma	161	11.51	16.53	23
6	Hodgkin lymphoma	177	31.8	13.59	44
7	Hodgkin lymphoma	182	11.26	1.00	14
8	Hodgkin lymphoma	195	90.80	44.28	10
9	Non-Hodgkin lymphoma	205	1.92	1.74	9
10	Hodgkin lymphoma	206	12.18	15.64	14
11	Ewing sarcoma	144	159.24	26.30	11
12	Ewing sarcoma	173	0.69	1.58	16
13	Ewing sarcoma	184	18.41	15.45	23
14	Ewing sarcoma	190	3.20	11.84	21
15	Bone marrow aplasia	152	14.68	15.12	45
16	Bone marrow aplasia	160	14.47	10.37	38
17	Myelodisplasia	152	4.07	11.26	17
18	Mixoide tumor	203	15.47	8.01	16
19	Thalassemia major	180	9.98	17.80	22
20	Thalassemia major	193	2.58	6.96	33
21	Hodgkin lymphoma	214	18.24	13.87	12
22	Systemic lupus Erythematosus	198	1.87	1.66	33

No. 1–4 premenarcheal patients, No. 5–22 postmenarcheal patients.

D/mm^2^: follicle density per mm^2^.

RO: right ovary; LO: left ovary.

**Table 3:** Characteristics of the post-chemotherapy patients who had ovarian tissue cryopreservation.

No.	Pathology	Chemotherapy	Age (months)	D/mm^2^ (RO)	D/mm^2^(LO)	Cryopreserved fragments No.
1	Wilms tumor	Vincristine, AMD	19	268.63	49.69	4
2	Wilms tumor	Vincristine, AMD	48	—	—	1
3	Acute myeloide leukaemia	ICE, AVE, HAM, ARA-C	70	112.84	56.23	19
4	Acute lymphoblastic leukaemia	Vincristina, cyclophosphamide, methotrexate	91	40.61	92.09	24
5	Acute lymphoblastic leukaemia	AIEOP 9502	170	12.03	22.62	67
6	Hodgkin lymphoma	COPP/ABV	81	59.38	—	10
7	Hodgkin lymphoma	COPP/ABV + IEP-OPPA	134	9.96	11.05	31
8	Hodgkin lymphoma	COPP/ABV	131	7.18	13.90	42
9	Hodgkin Lymphoma	ABVD	150	12.64	29.98	13
10	Hodgkin Lymphoma	COPP/ABV	165	5.66	8.87	55
11	Hodgkin lymphoma	COPP/ABV	166	19.19	43.34	14
12	Hodgkin lymphoma	COPP/ABV	175	5.55	10.81	13
13	Hodgkin lymphoma	IEP/IOPPA	177	30.33	31.57	21
14	Hodgkin lymphoma	COPP/ABV	197	6.80	11.99	8
15	Hodgkin lymphoma	COPP/ABV	215	5.08	7.67	49
16	Synovial sarcoma	IFO, AMD	154	15.12	14.95	47
17	Ewing sarcoma	ISG/SSG IV	170	8.04	7.82	71
18	Acute myeloide leukaemia	Idarubicin, etoposide, citrabina	180	3.67	4.56	20
19	Acute lymphoblastic leukaemia	Vincristine, daunomycin, predmisone	183	9.61	10.45	12
20	Acute lymphoblastic leukaemia	Unspecified	192	19.37	12.18	33
21	Acute lymphoblastic leukaemia	Vincristine, cyclophosphamide, methotrexate	209	12.02	11.36	53
22	Medulloblastoma	Vincristine	199	9.85	10.19	25
23	Peripheral neuroectodermal tumor	IFO, etoposide, thiotepa, adriamycin melphalan	212	0	0	61

No. 1–7 premenarcheal patients; No. 8–23 postmenarcheal patients.

D/mm^2^: follicle density per mm^2^.

RO: right ovary; LO: left ovary.

AMD: Actinomycin D; ICE: Idarubicin, Etoposide, Cytarabin; HAM: Mitoxantrone; ARA-C: Citosinarabinoside; AIEOP9502.

Protocol: Vincristine, Daunomycin, Predmisone, Asparaginase, Cyclophosphamide, 6-Mercaptopurine, Methotrexate, Adriamycin,

Dexamethasone, Cyclophosphamide, Citosinarabinoside, 6-Thioguanina; COPP/ABV: Vincristine, Procarbazine,

Cyclophosphamide, Prednisone, Adriamycin, Vinblastine, Bleomycin; IEP-OPPA: Ifofosfamide, Etoposide, Prednisone, Vincristine,

Procarbazine, Prednisone, Adriamycin; ABVD: Adriamycin, Bleomycin, Vincristine, Doxorubicin; IFO: Ifofosfamide; ISG/SSG IV.

Protocol: Vincristine, Adriamycin, Ifosfamide, Cyclophosphamide, Etoposide.
